# Optical coherence tomography visualization of stent deformation with subsequent thrombus adhesion at very early phase after everolimus-eluting stent implantation: a case report

**DOI:** 10.1186/s12872-016-0295-2

**Published:** 2016-05-31

**Authors:** Satoru Yamamura, Koichiro Fujisue, Kenichi Tsujita, Kenji Sakamoto, Yuji Miyazaki, Koichi Kaikita, Seiji Hokimoto, Hisao Ogawa

**Affiliations:** Department of Cardiovascular Medicine, Graduate School of Medical Sciences, Kumamoto University, 1-1-1 Honjo, Chuo-ku, Kumamoto City, 860-8556 Japan

**Keywords:** Optical coherence tomography, Thrombosis, Stent deformation

## Abstract

**Background:**

Stent malapposition, stent fracture, and deformity, and inadequate anti-thrombotic therapy are known as the risk of stent thrombosis. We report a case of stent deformation with subsequent thrombus adhesion at the site of a partial stent fracture detected by intravascular ultrasound (IVUS) and optical coherence tomography (OCT).

**Case presentation:**

A 61-year-old male patient was diagnosed as effort angina pectoris. Coronary angiography revealed obstructions in the proximal segment of the left anterior descending (LAD) and left circumflex artery (LCx). Elective percutaneous coronary intervention (PCI) was scheduled for these lesions in the prior hospital. First, an everolimus-eluting stent (EES) was implanted in the just proximal LAD slightly protruding into left main trunk. One week later, the treatment to residual obstruction at proximal LCx was attempted. During delivery of the stent to LCx, the proximal edge of the previously-implanted LAD stent got stuck with the newly-deploying stent and deformed into the intravascular lumen when retracting the stent into the guide-catheter. He was immediately transferred to our hospital to repair these procedural problems. Two days later after the index procedure to LCx, intracoronary imaging with an IVUS and OCT were performed, and the IVUS/OCT imaging revealed thrombus adhesion around the deformed struts. The three-dimensional OCT guide also helped the detection of the deformed stent and the repair of deformed struts by additional stenting and kissing balloon technique.

**Conclusion:**

The current case suggested that thrombus adhesion can occur at the site of deformed and/or fractured stent at very early phase after stent implantation.

**Electronic supplementary material:**

The online version of this article (doi:10.1186/s12872-016-0295-2) contains supplementary material, which is available to authorized users.

## Background

Drug-eluting stents (DES) reduce in-stent restenosis rate compared with bare-metal stents [1]. However, incomplete stent dilatation, stent fracture, deformity, and inadequate anti-thrombotic therapy are still known as the risk of stent thrombosis [2, 3]. Sent fracture has been reported in 1 to 2 % of patients after DES implantation, and that could cause stent thrombosis at early phase after stent implantation [4]. However, we might not always perceive the thrombus if the patients were asymptomatic, or no evidence of myocardial ischemia after the procedure of percutaneous coronary intervention (PCI).

Optical coherence tomography (OCT) is a technology which can capture micrometer resolution. OCT can provide real time and cross-sectional images of tissue structure including in situ thrombus. OCT could also detect stent fracture and early thrombus adhesion at the site of stent fracture [5]. OCT might prevent the coronary events complicated with early stent thrombosis.

Here, we report an interesting case of stent deformation with subsequent very early thrombus adhesion detected by intravascular ultrasound (IVUS) and OCT.

## Case presentation

**Fig. 1:**
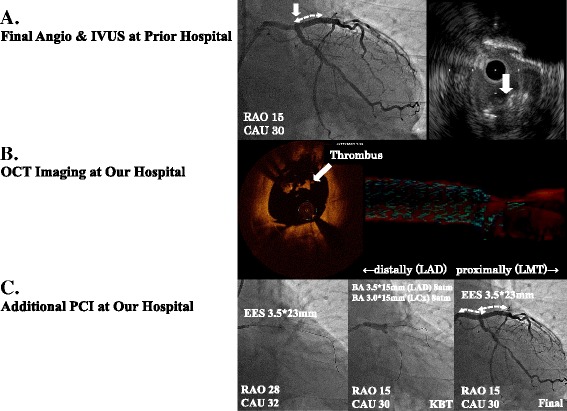
**a** Final left coronary angiography (CAG, right anterior oblique caudal view) at prior hospital did not show any significant obstruction from proximal left anterior descending coronary artery (LAD) to left main trunk (left panel). A stented segment is shown in white double-headed dashed arrow. Intravascular ultrasound (IVUS) image at just proximal LAD clearly demonstrated deformed stent struts protruding toward the lumen (white arrowhead), however, adhesion of thrombus to the struts was ambiguous on the IVUS image (right panel). The site of stent deformation was indicated by white arrow head on CAG (left panel). **b** Optical coherence tomography (OCT) imaging around the deformed struts performed at our hospital clearly showed thrombus adhesion to the deformed struts with radial signal attenuation behind the adherent structure to the struts (white arrowhead, left panel). 3D reconstruction image from proximal LAD to left main trunk (LMT) visualized fractured struts extending to the LMT direction shown in light blue (right panel). **c** Additional percutaneous coronary intervention underwent at our hospital. A 3.5x23mm EES was implanted from LMT to LAD (left panel). Kissing balloon technique using 3.5x15mm and 3.0x15mm non-compliant balloons were performed (middle panel). Final CAG after additional stenting followed by KBT (right panel). A stented segment is shown in white double-headed dashed arrow

A 61-year-old gentleman, with a history of hypertension and dyslipidemia and a habit of smoking, presented with effort chest pain. In the prior hospital, he was diagnosed as effort angina pectoris. A coronary angiography (CAG) detected 99 % stenosis at proximal left anterior descending artery (LAD) and 90 % stenosis at middle left circumflex artery (LCx). He was treated with optimal medical therapy including dual antiplatelet therapy with aspirin and clopidogrel as the guidelines recommend for prevention of secondary coronary events. He did not take any drugs that could influence the bioavailability of cropidgrel. Then, he underwent elective IVUS-guided PCI to proximal LAD. A 3.5 x 28 mm everolimus-eluting stent (EES) was implanted with excellent procedural success, as one strut length protruded to the ostium of LCx to completely cover the culprit lesion. One week later, he underwent elective PCI to LCx. However, this stent has stuck at the site of proximal edge of the LAD stent, when the operator tried to implant an EES to middle LCx. Pulling out this stent, however, the previously-implanted LAD stent was deformed into the left main trunk (Fig. 1a). The patient was transferred to our hospital to repair the stent deformity and complete the PCI procedure.

His vital sign was stable without any sing of angina or heart failure. Routine laboratory data were almost within normal limit. Electrocardiogram showed no ST-T changes. Transthoracic echocardiogram showed severely-decreased posterior wall motion with evidence of old myocardial infarction.

**Fig. 2:**
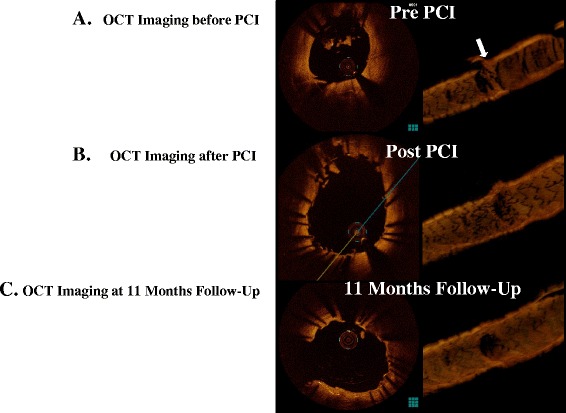
**a** Optical coherence tomography (OCT) image of the culprit lesion. 3D OCT showed deformed struts with adherent structure (white arrowhead, right panel), as in the cross-sectional image (left panel). **b** Post-PCI OCT imaging showed adequate stent expansion (left panel) and disappearance of the deformed struts with thrombus from the LMT bifurcation with widely-opened ostial circumflex (right panel). **c** OCT imaging at 11 months follow-up revealed nicely reendothelialized without restenosis. Thrombus was not observed at all

CAG was performed using a 6-Fr guide catheter via the right radial artery, and no significant obstruction was observed. A guidewire was easily advanced through the stent fracture site of LAD, and another guidewire was inserted into the LCx. For detailed morphological assessment of stent fracture, IVUS and OCT imaging were done. These modalities revealed a good apposition of the implanted stent, but a completely deformed stent with thrombus adhesion (Figs. 1b, 2a Additional file 1).

We decided to perform additional PCI to repair the stent deformity. A 3.5x23mm EES was deployed from the left main trunk (ostium) to middle LAD to completely cover the prior implanted stent. Postdilatation using 3.5x15 mm and 3.0x15 mm non-compliant balloons was performed at the bifurcation of LAD-LCx to attempt a kissing balloon inflation at the end of the procedure (Fig. 1c). After these procedure, we confirmed that there were not any coronary dissection and hematoma with adequate stent expansion using IVUS and OCT (Fig. 2b).

After this procedure, he did not experience chest pain and have no ST-T changes on the electrocardiogram. Eleven months later, we performed follow-up exercise test, CAG, IVUS and OCT to confirm whether myocardial ischemia and any structural problems of implanted stent occurred. There were no evidence of ischemia without restenosis and hematoma at that lesion (Fig. 2c).

## Discussion

Early stent thrombosis is closely related to high risk of myocardial infarction (MI) and mortality. The rate of stent thrombosis within 30 days is 1.4 % in patients with stable coronary artery disease [6]. There are no differences in the rate of early stent thrombosis between bare metal stents (BMS) and DES. Stent thrombosis is mainly caused by procedure-related factors such as insufficient inflation pressure, incomplete stent apposition, stent fracture (SF), dissection, and inadequate antiplatelet therapy [2, 3].

The incidence of SF of EES is 2.9 %. SF is associated with high risk of major adverse cardiovascular events [7]. The incidence of stent thrombosis after EES implantation is 0.3 % [8], and several cases of early stent thrombosis detected by IVUS and OCT have been previously reported [9, 10]. However, there was no report that demonstrated the thrombus adherence at early phase after EES implantation on the fractured stent struts by OCT.

Here, we first reported that OCT could detect the stent deformation with subsequent very early thrombus adhesion (3 days after PCI). With regard to OCT-guided PCI, generally, we have to check the mechanical complication such as edge dissection, stent underexpansion, or malapposition. In this case, as the patient complained of no chest pain, and CAG could not detect thrombus, if OCT or IVUS had not been employed, thrombus might become enlarged, and devastating myocardial infarction due to stent thrombosis could occurred. In such cases of stent fracture, we might have to check the existence of thrombus at the site of stent fracture by OCT, and repair the deformed stent as soon as possible if needed.

## Conclusion

Mechanical abnormality of coronary stenting and inadequate antiplatelet therapy can result in early stent thrombosis. OCT could be valuable to detect stent fracture and early thrombus adhesion to prevent serious subsequent complication such as myocardial infarction and sudden cardiac death.

## Abbreviations

BMS, bare metal stents; CAG, coronary angiography; DES, drug-eluting stents; EES, everolimus-eluting stent; IVUS, intravascular ultrasound; LAD, left anterior descending artery; LCx, left circumflex artery; MI, myocardial infarction; OCT, optical coherence tomography; PCI, percutaneous coronary intervention

## Additional file

Additional file 1: Three-dimensional movie of the deformed stent. (MPG 3234 kb)
